# The Effect of Real Ear Target Deviations on SSQ and Speech Intelligibility in a Clinical Population

**DOI:** 10.1177/23312165251408752

**Published:** 2026-01-19

**Authors:** Simon E. Lansbergen, Gertjan Dingemanse, Niek J. Versfeld, Wouter A. Dreschler, André Goedegebure

**Affiliations:** 1 Department of Otorhinolaryngology and Head and Neck Surgery, 639726 Erasmus MC University Medical Center Rotterdam, Rotterdam, The Netherlands; 2 Department of Clinical and Experimental Audiology, 26066 Amsterdam UMC, University of Amsterdam, Amsterdam, The Netherlands; 3 Otorhinolaryngology – Head and Neck Surgery, 541223 Amsterdam UMC Location VUmc, Amsterdam, The Netherlands

**Keywords:** NAL-NL2, REM, PROM, hearing aid, rehabilitation

## Abstract

The quality of hearing-aid (HA) fitting is typically evaluated using speech intelligibility tests and/or Real-Ear Measurements (REMs). Although it is assumed that a better fit improves daily outcomes, supporting evidence is inconclusive. This study examined whether deviations from National Acoustic Laboratories Non-Linear (NAL-NL2) real-ear targets (real-ear-to-target difference, RTD) predicted changes in Speech, Spatial, and Qualities of Hearing Scale (SSQ) scores, and whether they related to aided speech recognition in quiet. The effects of hearing loss and patient characteristics were also considered. Data from 298 adults (mean age 65 years) fitted with new or replacement HAs (66%) were analyzed. Baseline measures included unaided speech recognition in quiet and a 17-item SSQ; follow-up measures included aided speech recognition in quiet, RTDs, and the SSQ. Principal Components Analysis summarized RTDs into overall gain (RTD_1_) and high-frequency gain (RTD_2_). The effects of treatment, RTD, pure-tone average (PTA), audiogram slope, asymmetry, age, gender, and HA experience on SSQ scores were investigated with mixed-effects models. Hearing-aid use improved both SSQ score (by 1.4 points) and speech in quiet. The RTD_1_ predicted neither SSQ nor speech scores. Underamplification above 2 kHz (RTD_2_) did not influence speech scores significantly, but reduced SSQ improvement. Higher PTA and steeper slopes were associated with lower aided speech scores, while higher PTA and age reduced SSQ improvement. Hearing-aid experience showed modest SSQ-domain effects. About half of SSQ variance reflected between-subject differences. HAs provide substantial benefit, despite moderate NAL-NL2 mismatches. Accurate 4–8 kHz fittings maximize outcomes by the SSQ, supporting REM-guided fitting practices.

## Introduction

Hearing aids (HA) often offer a very suitable option for rehabilitation of a disabling hearing loss as they have a clear and proven positive contribution to reducing participation restrictions (Ferguson et al., 2017). However, this does not necessarily mean that HAs effectively address the communication needs of the users and resolve all participation restrictions due to the hearing loss, even if they are appropriately fitted to compensate for individual hearing loss. Therefore, an assessment of the patient's individual perception and needs is recommended (Munro et al., 2021).

Nowadays, patient-reported outcome measures (PROMs) are widely used instruments for validation and evaluation of a HA fitting (Abrams et al., 2012; Almufarrij et al., 2021, 2023; Cox et al., 2000, 2007; Dillon et al., 1997; Dornhoffer et al., 2020; Gatehouse & Noble, 2004) and have also found their way into mainstream hearing care (Lansbergen et al., 2018). Contrary to speech measures (e.g., word intelligibility in quiet), PROMs reflect experiences in different situations and hence are important indicators for the use of a HA (Cox et al., 2007; McCormack & Fortnum, 2013). Also, when administered prior to rehabilitation, PROMs increase the awareness of auditory (dis)ability by the individual, yielding clues for the practitioner that can increase the success rate of a HA fit (Cox et al., 2007).

While PROMs are considered a reliable validation measure, they do not offer detailed insights into the appropriateness of HA settings. In clinical practice, the quality of HA fitting is often examined using a Real-Ear Measurement (REM). The sound level measured near the tympanic membrane is compared with the output given by the so-called prescription rules, which prescribe gain or output based on hearing loss and patient characteristics. Two commonly used rules are the National Acoustic Laboratories Non-Linear (NAL-NL2; Keidser et al., 2012) and the Desired Sensation Level (v5.0; Scollie et al., 2005). Historically, manufacturer “first-fit” settings often deviate from these prescriptive targets (Abrams et al., 2012; Hawkins & Cook, 2003; Munro et al., 2016). Recent studies indicate that contemporary fitting software has improved initial fits, resulting in a modest median incremental change after REM-based fine-tuning. The largest difference between measured output and target typically occurs around 2–4 kHz, with wide inter-individual variability persisting (Almufarrij et al., 2023, 2024; Munro et al., 2021). Therefore, REMs remain important to quantify any real-ear-to-target difference (RTD). Although it is generally accepted that accurate fitting of HAs close to the desired prescriptive gain targets results in better auditory performance, the proven effect on patient-reported outcomes is limited (Almufarrij et al., 2021; Narayanan et al., 2023). In their systematic review, Almufarrij et al. (2021) found that only a few studies showed a statistically significant advantage of REM-based fittings. This resulted in a small overall effect of REM on self-reported benefit (about 4%). While earlier work reported preferences for REM-verified NAL fits over proprietary first-fits (Boymans & Dreschler, 2012), more recent studies indicate that, after REM-guided fine-tuning and incorporating patient feedback, the group-average improvement over initial fit is small and the reduction in target deviation is modest (Almufarrij et al., 2023, 2024). Furthermore, in a study by Narayanan et al. (2023) based on a large clinical population, it was shown that the total deviation of gain relative to the prescription rule was only partly related to perceived benefit of HAs, and dependent of the experience of users and the questionnaire employed. Overall, evidence that fitting HAs using REM has added value is sparse and inconsistent, reinforcing the need to understand how the deviation from target relates to individual outcomes.

Speech measures like word recognition in quiet or noise have been employed for many years in clinical settings to assess the effectiveness of HAs. Speech tests with words are used because they offer a controlled and practical measure of speech recognition. Although word tests do not fully represent the complexity of everyday listening, they provide a baseline measure of speech perception ability that correlates with communication in daily life situations. As such, these measures are in-between the verification of the fitting with REM and the validation with PROMs. Controlled studies indicate that REM-verified fittings that closely match prescriptive targets yield modest but significant improvements in aided speech recognition compared with manufacturer first-fits, particularly for soft speech and for sentences in noise (Humes et al., 2017; Valente et al., 2018; Venail et al., 2021). Also, the observed benefits can be highly dependent on the speech materials and listening conditions (Almufarrij et al., 2021; Miles et al., 2022). An evaluation is required as to whether the difference between the REM and target predicts patient-reported benefit.

The aim of this study was to determine whether the quality of the HA fit obtained by REM, as defined by deviations from the NAL-NL2 description rule, is related to speech intelligibility and self-reported outcomes. The primary outcome was a change in self-reported hearing ability from pre- to post-fitting; the secondary outcome was speech intelligibility in quiet (unaided vs. aided). We further examined whether pure-tone average, audiogram slope, age, prior hearing-aid experience, gender, and interaural asymmetry modified these relationships.

## Methods

### Subjects

This study was conducted at the Erasmus Medical Center (EMC) in Rotterdam, and relevant data were collected from clinical records between July 16, 2015, and October 30, 2019. This is a retrospective, consecutive cohort. The time window was chosen because the Speech, Spatial, and Qualities of Hearing Scale (SSQ) short form (see below) had been implemented as routine clinical practice at EMC; therefore, the final sample size reflects all eligible cases within this period after applying prespecified inclusion/exclusion criteria. No priori sample-size calculation was performed; instead, we included all consecutive eligible patients to maximize precision and avoid selection bias. We report effect sizes and confidence intervals to convey the precision of estimates. To be included in the study, subjects had to meet the following criteria: (1) they were eligible for a new or replacement HA, and (2) they were 18 years of age or older at the time of HA fitting. Subjects eligible for HA replacement had been HA users for at least 5 years. Exclusion criteria encompassed the use of nonconventional HAs or implants (e.g., cochlear Implants, bone conduction devices, contralateral routing of signals [CROS] HAs, unilateral fittings), or cases where essential data required for the study were missing from the clinical records. Out of 3,000 subjects initially considered, 298 met the inclusion criteria and had their data utilized for further analysis. Informed consent was obtained from all subjects.

We deliberately included both first-time and experienced HA users to reflect real-world clinical practice and because prior HA experience is known to influence prescriptive targets (e.g., NAL-NL2 recommendations), acclimatization, preferred gain, and patient-reported outcomes.

### Hearing Rehabilitation Process

The rehabilitation pathway followed a standardized protocol with fixed steps: baseline assessment at EMC, initial fitting by a HA dispenser, trial period, and evaluation at EMC with REM and aided speech audiometry. Most subjects visited EMC twice: once before the trial period and again at the end of the trial period. Only data from these two visits were included in the analysis; data from possible interim visits were excluded. Interim fine-tuning occurred at the dispenser as needed. Exact numbers/timing of dispenser visits were not consistently documented and hence not analyzed. Nevertheless, the number of visits, the time intervals between fine-tuning visits, and the specific adjustments needed varied between patients, in order to meet patient-defined goals.

Before their initial appointment, subjects were asked to complete a SSQ questionnaire, which served as a baseline measurement of their subjective hearing abilities. This questionnaire helped both the clinician and the subject to gain insight into challenging auditory situations, and to formulate rehabilitation goals and select HAs. At the start of the rehabilitation process: (1) the pure-tone thresholds were measured, (2) speech intelligibility was assessed, (3) rehabilitation goals were established, and (4) HAs were prescribed. The fitting and initial adjustment of the HAs was carried out by the HA dispenser chosen by the subject.

Subsequently, an evaluation appointment was scheduled at the medical center. In routine practice, this evaluation is scheduled several weeks after the initial fitting; because of the retrospective design, the exact intervals and the number of dispenser visits occurring in the interim were not systematically documented and are therefore not analyzed here. Prior to this appointment, subjects completed the SSQ questionnaire again. During the evaluation appointment, aided speech intelligibility and the amplification characteristics (using REM) were measured with the devices set to the participant's everyday-use settings as prescribed at the end of the trial period. Thus, participants completed the SSQ twice: pre (before the initial fitting) and post (immediately prior to the evaluation visit). If the rehabilitation goals were not met, additional appointments were scheduled with either the dispenser or EMC until both the subject and the clinician were satisfied. Throughout the entire process, the HAs were on trial and were only purchased after successful evaluation and fitting.

### Speech, Spatial, and Quality of Hearing Questionnaire

The original SSQ questionnaire was developed by Gatehouse and Noble (2004) to measure a range of hearing abilities in relation to the following domains: speech intelligibility (SSQ_Speech_), spatial hearing (SSQ_Spatial_), and the quality of sound (SSQ_Quality_). The SSQ contains 49 items (14 speech, 17 spatial, 18 quality). Subjects rated each item with a score between 0 and 10, that is, absolute ability ratings at a given time point. Higher scores reflect greater ability (i.e., less disability). At EMC an abbreviated Dutch version of the SSQ (called SSQclin) was developed and used in the rehabilitation process (Knoop et al., 2022). This clinical SSQ consists of a total of 17 items (7 speech, 3 spatial, and 7 quality) and was developed with the aim of having a short, clinically optimized SSQ. This clinical SSQ was developed from the perspective of following individual progression over time and to be able to monitor the quality of care at group level. There is no established baseline-specific minimal clinically important difference (MCID) for SSQclin. Our analyses therefore consider both absolute post-fitting scores and the within-subject change from pre- to post-fitting. We did not use the comparative or benefit versions of the SSQ (Jensen et al., 2009). For experienced users, the pre SSQ reflects everyday listening with their existing (older) device(s) prior to refitting; participants were not asked to provide retrospective “as if unaided” ratings. Analyses considered absolute post-fitting scores and within-subject pre- to post-change. Repeated measurements were handled with linear mixed-effects models including a subject-specific random intercept and a within-subject Treatment factor (pre vs. post).

### Pure-Tone Audiometry

Pure-tone audiometry was performed across a frequency range of 0.25–8 kHz, following the shortened ascending method as stipulated by ISO standard 8253-1 (ISO, 2010). All audiometric tests were conducted in a soundproof booth. The audiometers used for these tests were calibrated in accordance with ISO 389 (ISO, 2018). From the gathered pure-tone threshold data, several key metrics were derived:

1. *Pure-Tone Averages (PTA):* calculated as the mean of thresholds at 0.5, 1, 2, and 4 kHz frequencies for each subject and evaluated for both ears separately.
2. *Audiogram Slope:* determined by the average slope in decibels per octave (dB/oct) for thresholds between 1 and 4 kHz.
3. *Asymmetry Measure:* the absolute difference between the PTA of both ears.

### Speech Audiometry

Speech intelligibility was assessed using Dutch CVC wordlists presented in quiet (Bosman & Smoorenburg, 1995). All tests were completed in a soundproof booth. Measurements were conducted to evaluate both unaided and aided speech intelligibility under controlled conditions. Unaided speech intelligibility was first measured before any treatment. Using headphones, subjects were presented with speech stimuli at varying intensities, including levels just below the Speech Reception Threshold (SRT)—defined as the intensity level at which 50% of the speech material is correctly identified—as well as levels where maximum speech intelligibility scores could be obtained for each ear. The key measure for unaided speech intelligibility (SI_U_) was determined as the percentage of correctly repeated phonemes at an intensity level of SRT + 10 dB. This was done because a fixed level would have yielded floor effects for many participants with moderate-to-severe loss; SRT + 10 dB places testing near the steep region of each listener's psychometric function and provides a comparable, clinically meaningful point across ears and degrees of hearing loss (see also the close relation between SRT and PTA in Bosman and Smoorenburg, 1995). Aided speech intelligibility (SI_A_) was measured binaurally in the free field using speech stimuli presented at 55 dB SPL from a loudspeaker positioned one meter directly in front of the subjects (0° azimuth). This method enabled the evaluation of the HAs’ effectiveness in improving speech perception in a standardized auditory environment.

Note that, by design, SI_U_ and SI_A_ are obtained at different levels: SI_U_ at SRT + 10 dB (ear-specific) and SI_A_ at a fixed 55 dB SPL level (binaural). This combination was chosen to avoid floor effects in unaided testing and ceiling effects in aided testing, yielding complementary, clinically meaningful points of comparison across the hearing-loss range.

### Real-Ear Measurements and Prescription Rule

The assessment of the output of the fitted HAs was carried out through REMs. This involved measurement of the output of a HA as a function of frequency (Real-Ear Aided Response, REAR) using a small probe microphone placed in the ear canal near the eardrum (Dillon, 2008). The REAR measurements were obtained using the “Affinity” REM equipment by Interacoustics (Denmark) and subsequently converted to octave bands. Each octave frequency's REAR value represented the mean sound energy measured within the corresponding frequency band. To ensure accurate REAR measurements up to 8 kHz, the probe insertion depth was standardized at 31 mm for males and 28 mm for females, relative to the tragus (Vaisberg et al., 2016). An external loudspeaker (Genelec 8010) presented the International Speech Test Signal (Holube et al., 2010), at an input level of 65 dB SPL, at which the REAR measurements were acquired. The REARs were measured with the HAs in the listener's everyday program and habitual volume setting, that is, as worn in day-to-day use; no additional adjustments were performed at the measurement session. The HA type/model, receiver strength, and coupling (e.g., open vs. occluded/vented) were determined by the dispenser or clinician and were not standardized or systematically recorded. Consequently, achievable real-ear bandwidth, particularly at 4–8 kHz, likely varied across subjects. In routine clinical practice, high-frequency gain may also be intentionally curtailed in ears with very steep high-frequency loss or suspected cochlear dead regions to optimize comfort, sound quality, and feedback risk. The REAR data reflect a single post-fitting measurement in “as-worn” settings. No pre-fitting or pre-post REM comparison was available.

The REAR measurements were then compared to response targets prescribed by the NAL-NL2 (Keidser et al., 2012). This is an empirically derived HA prescription rule, which was utilized to compute response targets for the REAR measurements, primarily based on the degree and type of hearing loss. The NAL-NL2 also accounted for user-specific characteristics such as gender, language type, previous HA experience, and binaural loudness summation. In this study, response targets were determined for the “experienced user” setting, as all users eventually become experienced after a habituation period. Language type was set to “nontonal,” with other parameters adjusted according to the subjects’ gender and pure-tone audiogram. During the study period, a single numeric pass/fail tolerance for NAL-NL2 target matching was not mandated. Because uniform tolerance was neither enforced nor consistently documented, we analyzed the RTD as a continuous predictor rather than classifying fittings as “within” or “outside” tolerance.

#### Criteria for Ear Selection

For all ear-specific analyses, including hearing thresholds, data from the ear with the better PTA were used. In cases where both ears had equal PTAs, the right ear was selected. This approach was chosen to minimize variability associated with asymmetrical hearing loss and to standardize the assessment of HA effects. In some cases, REM data from the poorer ear were used when the better ear could not be fitted with a HA, or when technical or anatomical constraints prevented REM measurement on the better ear.

#### Goodness-of-Fit and Statistical Modeling with Principal Component Analysis

To analyze the aided response data measured at 65 dB SPL, we calculated the RTD by subtracting the NAL-NL2 target values from the measured REAR values. This RTD measure was computed for octave frequencies ranging from 0.25 to 8 kHz. Principal Component Analysis (PCA) was harnessed to effectively summarize the RTD data, yielding one or more fundamental RTD curves as a function of frequency, referred to as the principal components (PCs). Each individual subject's RTD curve could then be represented as a weighted sum of these PCs, with these weights known as loadings. This approach reduced the data's complexity, approximating each individual's RTD curve with a smaller set of values (the loadings) instead of the original six RTD values.

To assess the significance of these PCs and their correlation with the original RTD data, we employed a permutation test, following the methodology suggested by Vieira (2012). This statistical method allowed us to evaluate the relevance of each PC in accurately representing the RTD data.

### Statistical Modeling

The study utilized a linear mixed-effects (LME) modeling approach to analyze data from the SSQ and speech intelligibility assessments, collected at two distinct time points (pre- and post-treatment). All models included a subject-specific random intercept (1 | Id) to account for within-participant dependence of pre-post observations, with Treatment (pre vs. post) specified as the within-subject factor. This modeling approach efficiently manages complex data structures, such as repeated measures and hierarchical groupings, by accommodating inherent dependencies among multiple correlated responses within subjects.

The performance of the LME models was assessed by examining both Conditional and Marginal *R*² values. Conditional *R*² reflects the proportion of variance in the dependent variable explained by both fixed and random effects, while Marginal *R*² represents the variance explained solely by the fixed effects. These metrics provide insights into the model's ability to account for both the fixed predictors and the variability across subjects.

In the LME models, we standardized variables by scaling, converting them to a common scale with a mean of zero and a standard deviation (SD) of one (z-scoring). This normalization ensures that model coefficients are comparable and improves the stability and convergence of the model, especially when combining variables measured in different units. Accordingly, fixed-effect coefficients for predictors reflect the expected change in SSQ points per 1 SD change in the predictor. For clinical interpretability, we converted selected standardized effects back to raw units using observed SDs (e.g., PTA SD = 17 dB; Age SD = 14.8 years). We also applied a Rationalized Arcsine Unit (RAU) transformation to speech intelligibility scores to homogenize variance and normalize distribution, a common approach for managing bounded percentage scores (Studebaker, 1982).

#### The Impact of Hearing Aids on Speech Intelligibility

This analysis assessed the impact of HAs on speech perception in controlled conditions. The dependent variable, “Speech,” is the speech intelligibility score. Key factors in the model included “Treatment” (indicating pre- or post-treatment stage), “RTD” (derived from a PCA), “PTA,” and “Slope.” Participant characteristics such as “Age,” “Experience” (previous HA experience), “Gender,” “Asymmetry” (measuring the absolute difference in PTA between ears), and “Ear Fitted” (whether the HA was fitted on the best or worst ear) were also included. In this analysis, “Treatment” denotes unaided (pre) versus aided (post) measurement condition; it does not index a change in fitting approach or a REM-optimization step.

Model formula before stepwise elimination in R pseudo-code:

Speech∼Treatment*RTD*PTA+Slope+Age+Gender+Experience+Asymmetry+EarFitted+(1|Id)

The inclusion of Treatment * RTD * PTA in the model formula implies that all main effects, as well as two-way and three-way interactions between these variables, were considered. The random effect (1 | Id) was used to capture individual variability, adjusting for dependence of observations within the same subject.

#### Impact of Hearing Aids on SSQ Scores

The base model in R pseudo-code incorporated various fixed and interaction effects. The dependent variable, “SSQ Score,” from the SSQ questionnaire, was influenced by “Treatment,” “SSQ Domain” (representing the three subdomains of the questionnaire), “RTD,” and “PTA.” Other variables such as “Experience,” “Gender,” “Asymmetry,” and “Ear Fitted” were also part of the model. The SSQ was collected pre- and post-fitting; REMs were only available at post-fitting. Experience status was coded as a fixed factor and allowed to interact with SSQ domain and aided speech, so that any differential effects between user groups were estimated rather than confounded. Accordingly, pre–post change in experienced users indicates replacement benefit (new vs. previous device), whereas, in first-time users, it reflects the transition from unaided to aided hearing.

Model formula before stepwise elimination in R pseudo-code:

$$\begin{array}{rl} & \mathrm{SSQ}\;\mathrm{Score}\;∼\;(\mathrm{Treatment}\;*\;\mathrm{SSQ}\;\mathrm{Domain}\;+\mathrm{PTA}\;+\mathrm{Slope})\\ & \;\;\;*\;\mathrm{RTD}\;+\mathrm{SSQ}\;\mathrm{Domain}\;*\;((\mathrm{Age}\;+\mathrm{Experience})\;\\ & \;\;*\;(\mathrm{Aided}\;\mathrm{Speech}\;+\mathrm{PTA}\;\\ & +\mathrm{Slope})\;+\mathrm{Gender}\;+\mathrm{Asymmetry}\;+\mathrm{Ear}\;\mathrm{Fitted})\;+(1\;|\;\mathrm{Id}) \end{array}$$

To simplify our models, we employed an automated stepwise model selection process. This method involved starting with a comprehensive base model that included all plausible predictors and their interactions. The process then systematically evaluated each term's contribution. Using automated procedures, nonsignificant variables were progressively removed (backward eliminated), and the model's fit was continually reassessed. The stepwise selection was based on the Akaike Information Criterion, which penalizes model complexity while rewarding goodness of fit.

Following the stepwise model selection process, a Variance Inflation Factor (VIF) analysis was conducted, checking for multicollinearity among the retained variables, ensuring that highly correlated predictors did not undermine the reliability and interpretability of the regression coefficients. By confirming low VIF values (typically VIF < 5), we verified that the final models were free from significant multicollinearity, thus validating the independence and impact of each predictor.

For post hoc analysis and effect size estimation in the LME models, we employed (partial) omega squared (ω^2^), as it provides an accurate population effect size estimate. This choice is preferred in large samples where other measures, like eta-squared (η^2^), may overestimate the true effect size and may lead to biased conclusions (Field et al., 2012; Olejnik & Algina, 2003). In general, results were considered significant at a *p*-value of less than or equal to 0.05. However, we considered small or smaller effect sizes (as defined by Cohen's guidelines: small (ω^2^ = 0.01), medium (ω^2^ = 0.06), and large (ω^2^ = 0.14)) to be clinically irrelevant, as they likely do not signify meaningful differences in rehabilitation outcomes and could be negligible for practical purposes.

#### Software

All analyses were conducted using RStudio (RStudio Team 2024. RStudio: Integrated Development for R. RStudio, Inc., Boston, MA). The PCA analysis was conducted using the standard RStudio library. Statistical significance testing for the PCA was performed using the PCA test package by Camargo (2022). Linear mixed-effects models were executed using the lme4 package (Bates et al., 2015). For post hoc analyses, both the car package (Fox & Weisberg, 2019) and sjstats package (Lüdecke, 2024) were utilized.

## Results

### Subject Statistics and Pure-Tone Audiometry

The study included a total of 298 subjects (159 males, 139 females). The mean age of participants was 65.2 years, with a SD of 14.8 years. Prior experience with HAs was reported by 196 subjects (65.8%), while 102 subjects (34.2%) had no prior experience. The REM data were predominantly used from the better ear in HA fittings, accounting for 94.0% (280 subjects), whereas the REM data from the worse ear were used in the remaining 6.0% (18 subjects). The mean PTA at 0.5, 1, 2, and 4 kHz (PTA) was 49.4 dB HL with an SD of 17.0 dB. The relatively high mean PTA in our population reflects the referral patterns in Dutch healthcare, where patients with more complex or severe hearing losses are directed to specialized audiological centers. The PTA slope had a mean of 5.6 dB/octave with an SD of 5.5 dB/octave, and the mean asymmetry PTA was 11.2 dB with an SD of 10.9 dB (note that asymmetry PTA is always positive by definition). The mean speech-aided score at 55 dB SPL was 76.9%, with an SD of 21.0%.

PTA was moderately but significantly correlated with age (*r* = 0.313, *p* < 0.001), and a small but significant positive correlation was also observed between slope and age (*r* = 0.174, *p* = 0.003), indicating that older participants tended to have greater hearing loss and steeper high-frequency configurations.

### Real-Ear Measurements and Prescription Rule

**Figure 1.**
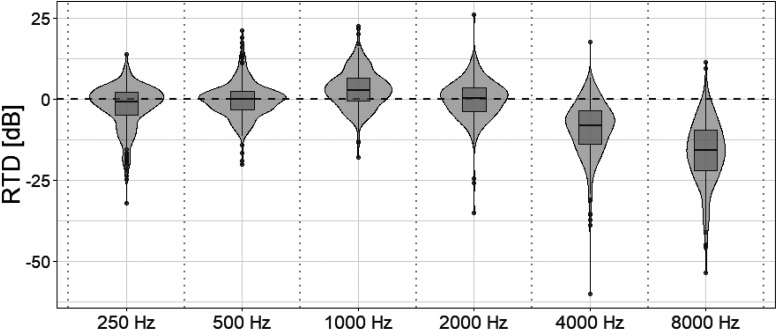
Combined box plot and density plot of real-ear-to-target difference (RTD) per octave frequency: from 0.25 kHz to 8 kHz. Positive RTD values indicate overamplification, while negative values represent underamplification with respect to the National Acoustic Laboratories Non-Linear (NAL-NL2) prescription rule.

Figure 1 provides an overview of RTD per octave frequency, for all 298 subjects. A negative RTD value implies underamplification with respect to the NAL-NL2 prescription rule. It can be noted that median values for the lower and middle frequencies, up to 2 kHz, were “on target.” In contrast, for the higher frequencies of 4 and 8 kHz, the target was often not reached, resulting in underamplification. Interestingly, the largest spread (i.e., variance) in the data was at 4 and 8 kHz.

Numerical summaries underline the under-amplification at high frequencies. At 4 kHz, the median RTD was −8.4 dB with an interquartile range (IQR) of −14.0 to −3.7 dB (range −60.2 to +17.5 dB). At 8 kHz, the median RTD was −15.9 dB with an IQR of −22.2 to −9.7 dB (range −53.7 to +11.4 dB). In contrast, medians at 0.25, 0.5, 1, and 2 kHz were close to target (−0.9, −0.1, +2.7, and +0.2 dB, respectively). A small number of extreme outliers (>50 dB under-target at 4–8 kHz) were retained to reflect real-world fittings; these likely reflect cases with severe high-frequency loss where targets exceed device/receiver or coupling bandwidth, open/vented couplings, and/or rare measurement artefacts, and they do not affect the reported medians or IQRs.

To benchmark these deviations against a commonly used clinical tolerance window of ±5 dB for average-level inputs across 0.25–6 kHz (BSA, 2018), we summarized medians and interquartile ranges (Q1–Q3) per frequency. At 0.25, 0.5, and 2 kHz, the medians were within ±1 dB (−0.9, −0.1, and +0.2 dB, respectively), and the IQRs lay entirely within ±5 dB (0.25 kHz: −5.0 to +2.1; 0.5 kHz: −3.3 to +2.4; 2 kHz: −3.8 to +3.4), indicating that at least half of fittings met the ±5 dB criterion at these frequencies. At 1 kHz, the median was +2.7 dB with Q3 at +6.3 dB, implying that at least one quarter of fittings exceeded +5 dB on the high side. At 4 kHz, the median deviation was −8.4 dB (Q1 −14.0, Q3 −3.7), indicating that at least half of fittings fell outside the ±5 dB window. At 8 kHz, the IQR was −22.2 to −9.7 dB with a median of −15.9 dB, meaning that at least 75% of fittings were outside ±5 dB at that frequency. We did not measure 6 kHz; nevertheless, the pattern indicates that tolerance exceedances become common above 4 kHz.

#### Real-Ear-to-Target Difference Profiles

**Figure 2.**
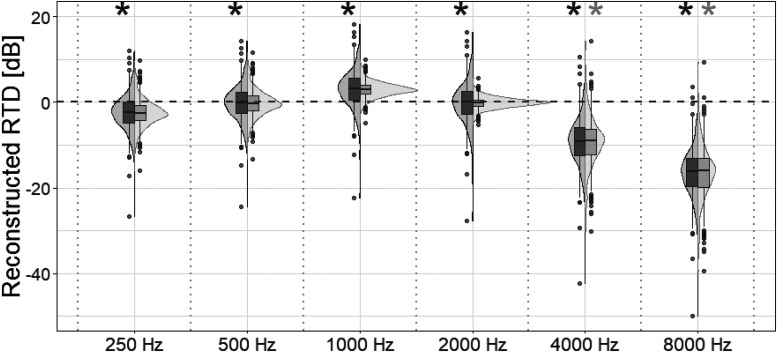
Combined box plot and density plot of real-ear-to-target difference (RTD) profiles reconstructed from the first two principal components (PC) that resulted from a Principal Component Analysis (PCA) of the original RTD data. RTD per octave frequencies: from 0.25 kHz to 8 kHz. RTD for the first reconstructed PC in dark gray, and the second PC in light gray. Asterisks at the top of each frequency indicate that PC loadings correlated significantly with original RTD data for that frequency.

A PCA applied to the RTD data resulted in a set of PCs that were tested for significance using permutation testing. The first 2 PC axes were significant (*p* < 0.001) and accounted for 77.9% of the total variance (51.2% and 26.6% variance for PC1 and PC2, respectively). These PCs will be referred to as RTD_1_ and RTD_2_. The RTD frequencies 0.25, 0.5, 1, 2, 4, and 8 kHz have significant (*p* < 0.001) loadings on RTD_1_, while frequencies 4 and 8 kHz loaded significantly (*p* < 0.001) on RTD_2_. Figure 2 shows a reconstruction of RTD_1_ (dark gray) and RTD_2_ (light gray) using the original RTD data. From Figure 2, it can be observed that RTD_1_ reflects overall variation in RTD—that is, the average difference between the REAR and the NAL-NL2 target, hence differences in overall gain between subjects. The boxplot illustrates a comparable spread in variation across all six frequency bands. In contrast, RTD_2_ captures variation specifically at the two highest frequencies (4 and 8 kHz). By design, RTD_1_ and RTD_2_ are uncorrelated, allowing both to be included simultaneously as independent variables in the LME analysis.

Linear regression models were used to examine the effects of age, hearing loss (PTA), and prior HA experience on RTD_1_ and RTD_2_. For RTD_1_, no significant effects were found for any of the predictors. In contrast, RTD_2_ was significantly influenced by PTA (*p* < 0.001, *f* = 0.52), with greater hearing loss associated with more negative RTD_2_ values, indicating larger deviations from the NAL-NL2 target at higher frequencies. Supporting this, correlation analyses showed moderate positive correlations between RTD_2_ and both PTA (*r* = 0.417, *p* < 0.001) and audiogram slope (*r* = 0.456, *p* < 0.001), whereas RTD_1_ showed weak but statistically significant negative correlations with PTA (*r* = −0.117, *p* = 0.044) and slope (*r* = −0.125, *p* = 0.030). These results suggest that fitting deviations at higher frequencies are systematically related to the degree and configuration of hearing loss.

### Impact of Hearing Aids on Speech Intelligibility

This analysis investigated the predictors of speech intelligibility with HAs using a LME model. Our goal was to understand how different predictors influence the outcome of speech intelligibility in an unaided (pretreatment) condition and an aided (post-treatment) condition. After stepwise elimination, the final model was formulated in R pseudo code as follows:

Speech∼Treatment+PTA+Slope+Treatment:PTA+(1∣Id)

#### Model Fit and Explained Variance

The Conditional *R*² was 0.435, indicating that 43.5% of the variation in speech intelligibility is explained by the combined fixed and random effects. This suggests a strong overall model fit that captures both individual variability and the influence of the predictors. The Marginal *R*² was 0.391, meaning 39.1% of the variance is attributable to the fixed effects alone. The relatively small difference between the two *R*² values indicates that individual differences contributed modestly to the overall model fit.

The Root Mean Square Error (RMSE) was 15.38 (RAU-score), reflecting the average deviation of predictions from observed values, with a lower RMSE indicating a reasonable model performance. Sigma was 16.02, closely aligned with RMSE, demonstrating consistency in the model residuals.

#### Fixed Effects

The fixed effects revealed significant predictors (see Table 1):

- *Treatment:* A positive estimate of 5.11 indicates a significant improvement in speech intelligibility following treatment, although the effect size is small (partial ω^2^ = 0.02).
- *PTA:* A positive estimate of 0.04 suggests that individuals with higher PTA (greater hearing loss) showed slightly greater improvements in speech intelligibility at baseline. The effect size was large (partial ω^2^ = 0.26).
- *PTA Slope:* A negative estimate of −1.56 indicates that a steeper high-frequency hearing loss was associated with reduced improvements in speech intelligibility when comparing aided and unaided conditions. The effect size for this predictor was small (partial ω^2^ = 0.01).
- *Treatment × PTA:* A significant negative interaction (estimate = −17.07) indicates that the benefits of treatment on speech intelligibility were reduced in individuals with higher PTA values. This interaction effect was large (partial ω^2^ = 0.23), suggesting that greater hearing loss limits the rehabilitative effectiveness of HAs at a fixed speech level of 55 dB SPL.

**Table 1.** Linear Mixed-Effects Model Results from the Speech Intelligibility Results Pre and Post Treatment, i.e., Hearing-aid Fitting.

Reference factor	Estimate (Beta) relative to reference	Std. Error	*t*-value	Partial ω^2^	*p*-value
(Intercept)	75.309	1.137	66.255		
Treatment	5.106	1.443	3.536	0.02	<0.001
PTA	0.044	1.119	0.039	0.26	<0.001
Slope	−1.559	0.760	−2.052	0.01	0.04
Treatment × PTA	−17.069	1.431	−11.927	0.23	<0.001

Reference Category; Treatment = Pre; ω^2^ ≥ 0.01 small, ≥0.06 medium, ≥0.14 large; PTA: pure-tone average.

The results (Table 1) show significant main effects of treatment and PTA on speech intelligibility improvement. However, these effects should be interpreted in light of their significant interaction: individuals with greater hearing loss (higher PTA) demonstrated slightly greater baseline improvements but benefited less from treatment compared to individuals with better hearing. Estimates are based on scaled variables and do not reflect the original units.

##### Notable Exclusions: RTD_1_ and RTD_2_

Initial model iterations included RTD_1_ and RTD_2_ as predictors, but they were excluded from the final model due to their insignificant contribution to the variance in aided speech intelligibility outcomes. As previously described under Figure 2, no significant association was found between RTD_1_ and hearing loss, while RTD_2_ showed a strong association with hearing loss. However, the variations in RTD captured by these components did not substantially influence aided speech perception in the context studied.

### Impact of Hearing Aids on SSQ Scores

In this study, we utilized a LME model to analyze data derived from the SSQ questionnaire. The objective was to evaluate the impact of RTD and various other factors on the SSQ scores. The final model formula, after stepwise elimination and VIF analysis, was formulated in R pseudo code:

$$\begin{array}{rl} & \mathrm{SSQ}\;\mathrm{Score}∼\mathrm{Treatment}+\mathrm{PTA}+{\mathrm{RTD}}_{1}+{\mathrm{RTD}}_{2}+\mathrm{Age}\\ & \;\;+\mathrm{Experience}+\mathrm{Aided}\;\mathrm{Speech}+\mathrm{Gender}+\mathrm{Asymmetry}\\ & \;\;+\mathrm{Treatment}:{\mathrm{RTD}}_{1}+\mathrm{Treatment}:{\mathrm{RTD}}_{2}+\mathrm{PTA}:{\mathrm{RTD}}_{2}\\ & \;\;+\;\mathrm{PTA}:\mathrm{Age}+\mathrm{Experience}:\mathrm{Aided}\;\mathrm{Speech}\\ & \;\;+\mathrm{SSQ}\;\mathrm{Domain}:\mathrm{Age}+\mathrm{SSQ}\;\mathrm{domain}:\mathrm{Experience}\\ & \;\;+\mathrm{SSQ}\;\mathrm{Domain}:\mathrm{Gender}+\mathrm{SSQ}\;\mathrm{Domain}:\mathrm{Asymmetry}+(1|\mathrm{id}) \end{array}$$

#### Model Fit and Explained Variance

The Conditional *R*² was 0.619, indicating that 61.9% of the variation in SSQ scores is explained by the combined fixed and random effects, reflecting a strong overall model fit. The Marginal *R*² was 0.301, meaning that 30.1% of the variance is attributable to the fixed effects alone. The substantial difference between the two *R*² values suggests that individual differences accounted for a considerable portion of the explained variance. The model's RMSE was 1.218 (points on the SSQ), and Sigma was 1.318, indicating consistency in the residuals of the model.

#### Fixed Effects and Interactions

The analysis of our mixed linear model yielded several insights into the factors affecting SSQ scores (Table 2):

- *Treatment Effect (pre- vs. post-treatment):* The treatment was associated with a significant improvement in scores (estimate = 4.52), with a very large effect size (partial ω^2^ = 0.25) signifying a significant benefit in terms of higher SSQ score from the HA intervention.
- *PTA Effect:* An increase in PTA, indicating worse hearing, was associated with a decrease in SSQ scores (estimate = −0.58), with a small effect size (partial ω^2^ = 0.04). This suggests that individuals with more pronounced hearing loss reported lower overall SSQ scores.
- *Age Effect:* Age showed a significant negative relationship with SSQ scores (estimate = −0.54), with a small effect size (partial ω^2^ = 0.02). As age increased, overall SSQ scores tended to decrease. This suggests that older individuals, compared to their younger peers with similar hearing loss, on average experience greater auditory disability, both before and after rehabilitation.

**Table 2.** Linear Mixed-Effects Model Results from the SSQ Questionnaire Pre and Post Treatment.

Reference factor	Factor relative to reference	Estimate (Beta) relative to reference	Std. Error	*t*-value	Partial ω^2^	*p*-value
(Intercept)		4.519	0.184	24.589		
Treatment	*Post*	1.432	0.063	22.707	0.25	<0.001
PTA		−0.575	0.148	−3.873	0.04	0.001
RTD_1_		0.028	0.054	0.512	<0.01	0.530
RTD_2_		0.115	0.087	1.315	<0.01	0.438
Age		−0.536	0.099	−5.433	0.02	<0.001
Experience	*Experienced*	−0.246	0.206	−1.194	<0.01	0.436
Aided Speech		0.031	0.168	0.183	<0.01	0.361
Gender	*Female*	0.022	0.180	0.123	<0.01	0.936
Asymmetry		−0.042	0.136	−0.311	<0.01	0.013
Treatment × RTD_1_		−0.119	0.040	−2.962	<0.01	0.003
Treatment × RTD_2_		−0.184	0.056	−3.279	0.01	0.001
PTA × RTD_2_		0.200	0.067	2.978	<0.01	0.003
PTA × Age		−0.217	0.102	−2.136	<0.01	0.033
Experience × Aided Speech		0.110	0.191	0.578	<0.01	0.564
SSQ Domain × Age	*Quality × Age*	0.113	0.082	1.371	0.01	0.047
	*Spatial × Age*	0.203	0.082	2.468		
SSQ Domain × Experience	*Quality × Experience*	0.666	0.125	5.345	0.12	<0.001
	*Spatial × Experience*	−0.403	0.125	−3.233		
SSQ Domain × Gender	*Quality × Gender*	0.325	0.141	2.309	0.01	<0.001
	*Spatial × Gender*	−0.353	0.141	−2.508		
SSQ Domain × Asymmetry	Quality × Asymmetry	−0.274	0.112	−2.439	0.01	<0.001
	Spatial × Asymmetry	−0.494	0.112	−4.399		

ω^2^ ≥ 0.01 small, ≥0.06 medium, ≥0.14 large

Reference Category; Treatment = Pre, SSQ = Speech domain, Experience = First User, & Gender = Male; RTD = real-ear-to-target difference; SSQ = Speech, Spatial, and Qualities of Hearing; PTA: pure-tone average.

Interactions of Interest:

- *Treatment × RTD_2_:* The interaction between treatment and RTD_2_ presented a significant negative estimate (estimate = −0.18), with a small effect size (partial ω^2^ = 0.01). This suggests that higher deviations from the target HA settings at the higher frequencies are associated with less improvement in SSQ scores after treatment.
- *SSQ Domain × Age:* There was a significant interaction effect between the domain of SSQ and age, with a small effect size (partial ω^2^ = 0.01). The effect was largest for the spatial domain (estimate = 0.20). This interaction should be interpreted as an addition to the negative effect of age on its own, as described above. The positive estimates suggest that the decline of age on the SSQ scores was less severe for the domains spatial and quality, compared to the effect on the speech domain (which was the reference).
- *SSQ Domain × Experience:* There was a significant positive interaction between the quality domain of SSQ and experience (estimate = 0.67), indicating that experienced users reported substantial higher overall SSQ scores in the quality domain, with respect to the speech domain. Conversely, the interaction for the spatial domain and experience was negative (Estimate = −0.40), suggesting that experienced users reported less overall spatial ability than inexperienced users. This effect was medium (partial ω^2^ = 0.12).
- *SSQ Domain × Gender:* The interaction between the quality domain of SSQ and gender showed that females reported higher overall perceived quality compared to males (estimate = 0.33), with a small effect size (partial ω^2^ = 0.01). In contrast, for the spatial domain, females experienced a significantly lower spatial ability compared to males (Estimate = −0.35).
- *SSQ Domain × Asymmetry:* The interaction effect between the quality domain and asymmetry demonstrated a significant negative association (estimate = −0.27), indicating that individuals with asymmetrical hearing loss perceived a lower auditory quality compared to individuals with symmetrical hearing (partial ω^2^ = 0.01), which falls into the small effect size category. A more pronounced negative interaction was observed for the spatial domain and asymmetry (estimate = −0.49), with a small effect size (partial ω^2^ = 0.01). This suggests that asymmetry in hearing significantly impacts the perceived spatial auditory abilities, leading to substantially lower scores in the quality domain.

Treatment significantly improved SSQ scores, with individual differences further shaped by hearing loss (PTA), age, HA fitting accuracy (RTD_2_), user experience, gender, and hearing asymmetry. Notably, higher PTA and older age were associated with lower overall SSQ scores. Deviations from target amplification at high frequencies (RTD_2_) reduced treatment benefits. Experience and gender selectively influenced perceived quality and spatial domains, while asymmetrical hearing loss negatively impacted both perceived quality and spatial abilities.

To aid interpretation in the SSQ's native units (0–10), selected standardized effects were converted to raw units. Worse hearing (PTA) was associated with ≈0.34 SSQ points lower per +10 dB (*βz* = −0.575; 10/17 SD). Age was associated with ≈0.36 points lower per decade (*βz* = −0.536; 10/14.8 SD). The Treatment × RTD_2_ coefficient (*βz* = −0.184) indicates ≈0.18 SSQ points less pre–post improvement per 1 SD worse high-frequency target match; across the interquartile range of RTD_2_ this corresponds to ≈0.25 points difference in improvement.

### Relationship Between Speech Intelligibility and SSQ

In this analysis, we explored the relationship between speech intelligibility, expressed in Rau scores for aided speech at 55 dB SPL, and SSQ scores in the speech domain. We utilized a linear regression approach, yielding the relationship:

y(x)=4.55+0.016x

**Figure 3.**
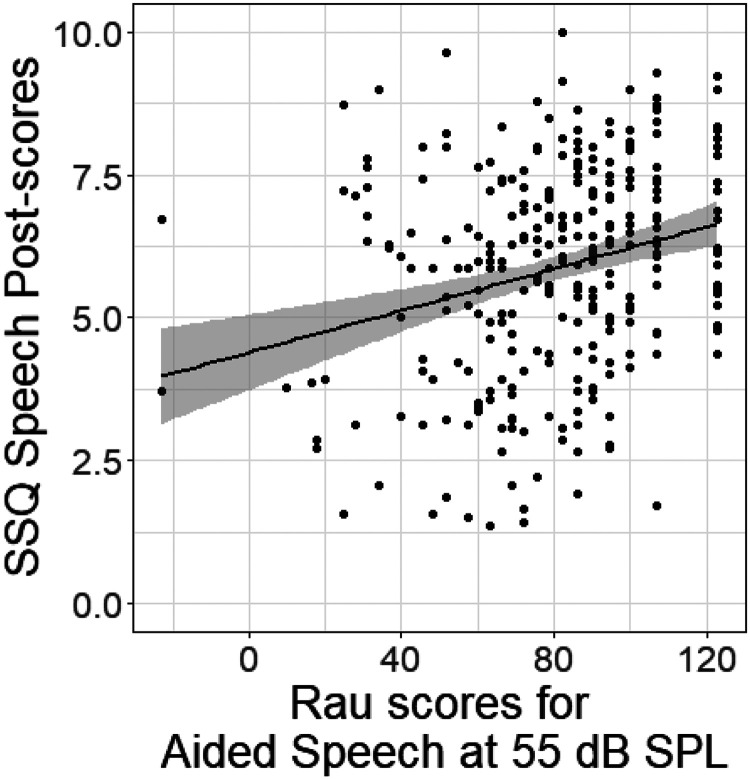
Scatter plot of Rau scores for aided speech at 55 dB against Speech, Spatial, and Qualities of Hearing (SSQ) speech post-scores with linear regression fit indicating a moderate correlation.

Here, *y* represents the SSQ Speech post-scores for the speech domain, and *x* denotes the Rau scores for aided speech. The model produced a coefficient of determination (*R*²) of 0.058 (*p* < 0.001). This indicates that approximately 6% of the variance in SSQ Speech post-scores can be explained by the level of aided speech intelligibility in quiet environments (Figure 3).

## Discussion

In a real-world clinical cohort, we observed three main patterns. First, new or replacement HAs yielded clear improvements in both aided speech in quiet (SI_A_) and SSQ, consistent with prior work showing benefit on objective and self-reported outcomes (Jensen et al., 2009; Lelic et al., 2024; Miles et al., 2022). Second, deviations from NAL-NL2 targets at 4–8 kHz (RTD_2_) showed a small but reliable association with self-reported improvement. Neither RTD_1_ nor RTD_2_ predicted SI_A_, which aligns with reviews and trials that have found modest and inconsistent average effects of REM on PROMs, as well as small-to-moderate effects on speech measures depending on materials and levels (Almufarrij et al., 2021, 2023, 2024; Narayanan et al., 2023). Third, configuration (slope) and severity of hearing loss (PTA) shaped outcomes: audiogram slope modestly constrained SI_A_, while higher PTA and age were linked to lower SSQ. Taken together, these findings suggest that high-frequency audibility should be considered during verification, at least in the present population.

In the next sections, these results will be discussed in more detail, from the perspectives of the different outcome measures used in this study.

### Real-Ear Measurements

The deviation from the NAL-NL2 targets at the higher frequencies in this study was somewhat larger than reported in recent cohorts of new HA users with milder losses, where incremental changes after REM fine-tuning over initial fit were modest and also concentrated within the 2–4 kHz range (Almufarrij et al., 2023, 2024). This discrepancy likely reflects differences in cohort composition, as our clinical sample included a broader range of hearing loss and steeper losses. This is also supported by the strong correlation we found between RTD_2_ and hearing loss. Greater hearing loss leads to increased underamplification at higher frequencies. In addition, our REMs represented “as-worn” settings at the time of evaluation rather than settings after REM-guided fitting to targets.

The deviation from the NAL-NL2 targets at higher frequencies may be attributed to at least three factors. First, similar to the study by Narayanan et al. (2023), the initial fit of the HAs in our study was not necessarily based on the NAL-NL2 fitting target. In many cases, it was a manufacturers’ proprietary fitting target. Much amplification of the high frequencies generally is not preferred by HA users (Nelson et al., 2018), likely because high-frequency sounds have often not been heard or have been heard less for years. In addition, the dynamic range is often small at higher frequencies. Manufacturers have the objective that their HAs should produce a comfortable sound right from the start, which is achieved by reducing the amplification of higher frequencies (Munro et al., 2016; Valente et al., 2018). Second, with larger hearing losses, the HA may be unable to provide sufficient gain at the higher frequencies due to constraints in the device (e.g., receiver/coupling bandwidth) or feedback limits; in routine fittings this often precludes substantial “usable” real-ear amplification at 8 kHz for a 65 dB SPL input. This effect of underamplification for frequencies above 2 kHz was also observed by Narayanan et al. (2023). Third, clinicians may intentionally limit high-frequency gain with very steep high-frequency slopes or suspected cochlear dead regions to balance audibility, sound quality, comfort, and feedback risk. We did not collect formal dead-region assessments or the dispensers’ fitting rationales, so we cannot attribute individual cases. Probe-tube insertion depth was standardized to support accurate measurement up to 8 kHz (Vaisberg et al., 2016), making systematic measurement errors unlikely.

Despite NAL-NL2's differing gain recommendations for first-time versus experienced users, we observed no RTD differences between groups, consistent with Thrailkill et al. (2019) and Narayanan et al. (2023). This likely reflects relatively lower PTAs in our first-time users, as the NAL-NL2 gain differences between first-time and experienced users only apply for PTA values above 40 dB HL (Keidser et al., 2011).

Taken together, these patterns point to high-frequency optimization (4–8 kHz) as the most practical verification target in routine care, even when exact matching is limited by bandwidth and feedback constraints.

### Speech Intelligibility

Hearing-aid usage significantly improved speech intelligibility at 55 dB SPL, though the size of this improvement decreased with increasing PTA. At this soft presentation level, performance typically remained below the ceiling. This pattern likely reflects the limited compensation offered by the NAL-NL2 half-gain rule at low input levels, resulting in lower aided scores for individuals with greater hearing loss. Importantly, this finding is influenced by our definition of SI_U_ as SRT +10 dB. Had we used unaided speech scores at a fixed 55 dB SPL level (as in standard audiometry), a positive relationship with PTA might have emerged, since those with more severe loss typically show worse unaided scores and thus greater potential for improvement. This methodological choice likely influenced the observed interaction and should be considered when interpreting the results.

Besides “Treatment” and “PTA,” the factor “Slope” showed a small but significant negative effect. Since SI_U_ was defined at SRT +10 dB, steeper audiograms yield shallower psychometric functions; hence, SI_U_ decreases as the slope increases. This finding is clinically relevant as it suggests that audiogram slope, not just overall hearing level, independently impacts auditory processing. Slope-related configurations may reduce spectral and temporal resolution, as discussed by Lorenzi et al. (2006).

Although age did not independently predict aided speech at 55 dB SPL when the PTA and slope factors were included, this does not imply that aging has no impact on listening. Age is closely tied to configuration (slope) and to suprathreshold factors that are not captured by a quiet, low-level speech measure, such as reduced temporal fine-structure processing and increased listening effort demands (Humes, 2002; Lorenzi et al., 2006). In other words, at 55 dB SPL, the audibility limitations reflected by PTA and slope largely determine aided performance, whereas age-related central or cognitive contributions are more likely to emerge in complex listening (e.g., speech in noise), which we did not measure here.

The RTD was not associated with enhanced speech recognition, a finding that may be attributable to at least three possible factors. First, as speech in quiet at 55 dB SPL depends largely on low- and mid-frequency audibility, the limited variation in RTD for frequencies below 2 kHz likely constrained any association between RTD and aided speech. Second, speech scores have a relatively low test–retest reliability (Carney & Schlauch, 2007), which may have added noise to the data that contributed to the absence of a significant relation between RTD and aided speech score. Third, the lack of RTD effects on SI_A_ likely reflects shared variance with PTA and Slope. The RTD_2_ was moderately correlated with both, and RTD_1_ showed weaker but significant associations. Thus, the predictive value of REM-based fitting quality—particularly at high frequencies—may already be captured by audiometric variables in this model.

Overall, these results reinforce the value of tracking both verification and validation as low to midfrequency audibility largely constrains quiet-speech performance at soft levels, whereas REM captures a broader perspective of performance and quality by covering the full frequency spectrum of the HA fitting.

### Speech, Spatial, and Qualities of Hearing Scores

Beyond a general improvement in self-reported auditory ability due to treatment, older age and higher PTA were associated with lower SSQ scores. Domain interactions suggested that effects of age-related decline were most pronounced for the Speech domain while the effects were smaller for the Spatial and Quality domains. This should be interpreted as a smaller decrease in those domains rather than improvement with age. These results suggest that older individuals and those with greater hearing loss reported more difficulties in everyday listening situations, even after rehabilitation. These associations should be interpreted bearing in mind the composition of the user population, which includes a wide range of hearing losses and ages. First-time HA users rated sound quality lower than experienced users, likely due to their lack of familiarity with amplified sound, while experienced users reported lower spatial abilities, which may be attributed to their greater degree of hearing loss. Asymmetry negatively affected both Spatial and Quality scores, consistent with its known impact on sound localization and auditory distortions in the worse ear (Kim et al., 2021; Zheng et al., 2022). These domain-specific effects were small-to-moderate and are best used to guide counseling rather than HA fitting.

Interpretation of PROM change must be anchored to the baseline. Analyses of large Handicap Inventory for the Elderly questionnaire datasets show that HA benefit scales with the unaided baseline. It is preferable to use baseline-specific “minimally detectable differences” and “MCIDs” thresholds rather than fixed cut points (Humes et al., 2025). Consistent with (Lansbergen, 2023, Ch. 8.), we therefore avoided single-value SSQ thresholds and modeled repeated measures (pre vs. post) rather than relying solely on raw difference scores and contextualized magnitudes in raw SSQ points and effect sizes. A published, baseline-specific MCID for SSQ short forms is not yet available. Our findings should thus be understood as statistically robust and clinically modest in size, pending SSQ-specific anchor-based work. Approximately half of the variance in SSQ scores was attributable to between-subject differences, which may reflect underlying psychological, or personality traits not directly measured in this study. Huang et al. (2017) demonstrated that personality characteristics are associated with perceptions of health-related quality of life. More specifically in audiology, Nordvik et al. (2021) found that higher levels of neuroticism were significantly associated with increased self-perceived hearing disability. These findings suggest that individual psychological profiles may substantially influence how hearing ability is perceived, independent of audiometric or demographic factors.

The SSQ norm scores for individuals with normal or impaired hearing are available (von Gablenz et al., 2018). The German SSQ short form was used to determine these norm scores, which is an adapted and abbreviated version of the SSQ (Kiessling et al., 2011). Although the SSQ items of their questionnaire do not exactly correspond to the items of the SSQ version used in this study, it does give a good impression of the range and variation for summarized SSQ scores. The results of von Gablenz et al. (2018) showed that 48% of all HA users scored lower than the 0.1 quantile of normal hearing subjects. Adjusted for age and HA use, our SSQ post-scores are comparable to these norm scores between all three domains and may therefore be considered as a representative result.

The RTD_2_ (high-frequency amplification) had a small but significant effect on SSQ scores: greater underamplification at 4 and 8 kHz was linked to poorer self-reported outcomes. Practically, this points to 4–8 kHz audibility as a modest but actionable lever for perceived benefit. Almufarrij et al. (2021) summarized the results of several other studies that found a relation between REM and improvement on self-reported listening ability. A trend seems to be present, but not all studies found evidence and effects were small. For example, Valente et al. (2018) and Narayanan et al. (2023) found significant SSQ advantages for REM-programmed fittings over initial fittings. Our findings differ in that we observed a small association between high-frequency RTD and SSQ change; however, our observational design did not test a REM-optimization intervention. Keidser et al. (2012) investigated the variability of individual preferred listening levels and reported a range of about 15 dB for all degrees of hearing loss. This could explain why overall amplification (i.e., RTD_1_) had no statistically significant effect on SSQ. Consequently, it seems appropriate to take into account and give room to individual preferred listening levels when fitting a HA, while striving in general for a fit that matches the shape of the NAL-NL2 target curve.

Despite the statistically significant trend suggesting that aided speech intelligibility generally correlates with SSQ, we observed a considerable spread in SSQ Speech post-scores, even at higher levels of intelligibility. This variation indicates that many individuals still experience a substantial amount of auditory disability, despite an adequate speech intelligibility performance in quiet. This may reflect personality traits as discussed before, but in addition it suggests that external factors such as cognitive processing, listening effort, and situational complexities (speech in noise) may influence self-reported auditory function beyond what is captured in a test of speech intelligibility in quiet with CVC-words.

### Strengths and Limitations

The novel contribution of this study lies in its systematic analysis of the relationship between REM data (defined as deviations from NAL-NL2 targets) and both patient-reported outcomes and speech intelligibility in a large clinical dataset. Few previous studies have used PCA to characterize fitting patterns across the frequency spectrum or examined these relationships in a population that includes both first-time and experienced users with such detailed REM data.

This was a retrospective, single-center study conducted in a tertiary audiology clinic, which enhances real-world relevance but may limit generalizability. In the Netherlands, more complex or atypical cases are referred to audiological centers, so our cohort likely had steeper losses and larger asymmetries than community samples. Hearing-aid selection and initial programming (often proprietary targets), whether and how REM was used during the trial, and interim fine-tuning were not standardized or fully documented. These uncontrolled factors add variability to the data, but they also reflect real-world conditions. As a result, the findings are highly relevant for clinical practice, as they capture outcomes under conditions that closely resemble standard care. We did not directly compare manufacturer initial fits with REM-optimized refits, and REMs were only available at the evaluation.

This study included data from REAR measurements at 65 dB SPL but did not include data for REAR at 55 dB SPL and/or 75 dB SPL, which can be considered as a limitation, since NAL-NL2 is a nonlinear prescription rule. Additionally, unaided SI_U_ and aided SI_A_ were obtained at different presentation levels. While this precludes level-equated change scores, it prevented major floor/ceiling artifacts and aligns with clinical priorities for assessing audibility of soft conversational speech. As noted in “Results,” this choice likely contributed to the observed Treatment × PTA interaction. This means that results presented in this study may not necessarily translate correctly to other sound levels. Finally, MCIDs for the SSQ short form are not yet established, so we interpret statistically robust effects as modest in clinical magnitude.

### Clinical Considerations

In relation to a commonly used ±5 dB clinical tolerance for average-level inputs (0.25–6 kHz) (BSA, 2018), our fittings generally met tolerance at low-to-mid frequencies (≤2 kHz), while exceedances were frequent at frequencies ≥4 kHz (Figure 1). This pattern likely reflects bandwidth and feedback constraints and the pragmatic clinical choice to limit very high-frequency gain. Because high-frequency cues contribute to clarity and speech understanding in more complex listening, targeted strategies to improve audibility at 4–8 kHz (e.g., feedback management, venting control, receiver upsizing, or frequency-lowering where appropriate) may yield incremental benefit. The ±5 dB benchmark is provided as a pragmatic context rather than a definitive standard.

Some of the results presented in this study could be considered helpful and of practical use by HA professionals in the field of audiology, these are summarized here:

- Aided speech measures and PROMs (e.g., SSQ) capture complementary aspects of rehabilitation. Using both provides a more complete picture of benefit progress.
- A better match to target for high-frequency amplification (>2 kHz) is associated with improved self-reported outcomes on the SSQ in this clinical cohort with predominantly moderate–severe loss. It did not predict speech intelligibility in quiet, but high-frequency audibility may matter more in complex listening (e.g., speech-in-noise, spatial hearing).
- Moderate deviations from prescriptive targets exert minimal influence on outcomes. Therefore, it is recommended to prioritize individualized adjustments while approximating target shape rather than overall gain.
- Differences between first-time and experienced users in perceived benefit are not fully explained by audiometric factors. First-time users tended to report lower sound quality, likely due to unfamiliarity with amplified sound. This highlights the importance of tailored counseling and adaptation support for new users.

## Conclusion

HAs significantly improve both speech intelligibility and self-reported hearing ability. However, the degree of benefit varies with the degree of hearing loss and high-frequency fitting accuracy. Our findings confirm that these improvements are evident not only in controlled measures but also in patients’ own perceptions of daily-life listening. High-frequency target matching (captured by RTD_2_ at 4 and 8 kHz) showed a small but reliable association with SSQ improvement, whereas it did not predict aided speech in quiet; thus, achieving an adequate response at and above 4 kHz may contribute to perceived benefit, but its clinical impact is modest and must be balanced with device bandwidth and individual preferences. Audiogram slope and other configuration factors also play an independent role in determining outcome, expanding the predictive model beyond PTA alone. In routine practice, ±5 dB matching was readily achieved at frequencies ≤2 kHz but less so for frequencies ≥4 kHz, highlighting the need for ongoing high-frequency optimization.

Auditory functioning with HAs is greatly influenced by initial performance, indicating that many factors, other than the amount of amplification, are also involved in auditory functioning with hearing loss. The findings highlight the need for individualized HA fitting strategies that balance prescriptive accuracy with user preferences, particularly for high-frequency amplification. Taken together, our results provide supportive evidence for the role of REM in guiding fittings, especially at high frequencies, where closer alignment to prescriptive targets was associated with improved self-reported benefits. While effects on speech intelligibility in quiet were limited, accurate high-frequency amplification may contribute to more positive experiences in everyday listening situations.
